# Coffee With Milk Stains and Attention Deficit Disorder

**DOI:** 10.1192/j.eurpsy.2026.11155

**Published:** 2026-07-31

**Authors:** M. P. Pando Fernández, P. Martinez Gimeno, M. Calvo Valcárcel, M. A. Andreo Vidal, M. D. L. Á. Guillén Soto, G. Guerra Valera, A. San Roman Uria, M. A. Parrilla Escobar, P. Marqués Cabeza, G. Lorenzo Chapatte, M. Rios Vaquero, L. Rojas Vazquez, A. Monllor Lazarraga, L. Sobrino Conde, L. Del Canto Martinez, F. J. Gonzalez Zapatero, E. Muñoz Ayuso, I. Larrañaga Gutierrez De Piñeres, D. Garcíamedall Arranz, M. E. Espinosa Muth, P. Anton Machado, S. Díez González

**Affiliations:** Psychiatry, HCUV, Valladolid, Spain

## Abstract

**Introduction:**

Neurofibromatosis type 1 (NF1) is a multisystem disorder characterized by multiple café-au-lait macules, intertriginous freckles, multiple cutaneous neurofibromas, and learning or behavioral problems. NF1 is inherited in an autosomal dominant manner. Penetrance is close to 100% (1).

Diagnoses of attention deficit hyperactivity disorder (ADHD) were more common in children with neurofibromatosis type 1 (NF1) than in the general pediatric population (2).

**Objectives:**

We present the case of an 8-year-old girl with NF1 and ADHD, highlighting the importance of early multidisciplinary intervention.

**Methods:**

We conducted a literature review by searching for articles in PubMed.

**Results:**

An 8-year-old female patient lives with her parents and 3-year-old sister. She is in the second year of elementary school.

Medical and surgical history: Neurofibromatosis type 1.

Mental health history: She has been attending outpatient appointments at Child and Adolescent Psychiatry since January 2023 for ADHD with a predominance of attention deficit.

Neuropediatric physical examination: Multiple café-au-lait spots and known ephelides. No neurofibromas (Image 1). Rest normal.

Psychopathological examination: Notable impairment in fine and gross motor skills. Fear of the dark. Behavioral and verbal impulsivity. Rest normal.

Complementary tests:

Brain MRI (2022): Bilateral T2 hyperintense lesions are seen in the globus pallidus, left thalamus, more tenuous in the midbrain, pons, and both cerebellar hemispheres. Compared to the MRI from May 2021, no changes are observed.

Other relevant examinations by different specialists show no notable alterations.

Diagnoses:

- NF type 1 without current neurological comorbidity.
- 314.01 (F90.2). Combined ADHD.
- Other neurodevelopmental difficulties (psychomotor skills)

Treatment: Modified-release methylphenidate 20 mg, one tablet with breakfast; psychotherapy.

Evolution: Significant improvement in different areas.

**Image 1:**
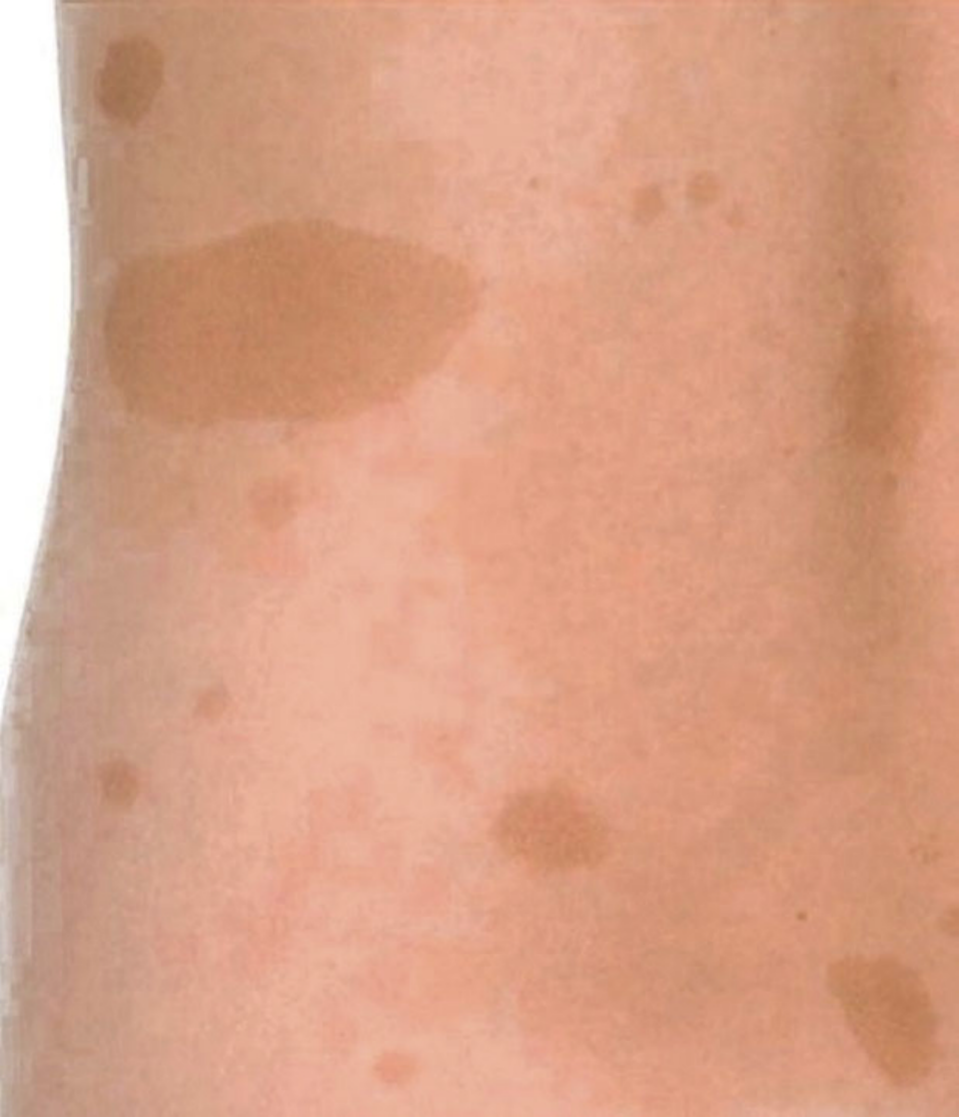

**Conclusions:**

According to the scientific literature reviewed, patients with neurofibromatosis type 1 (NF1) have a high predisposition to developing attention deficit disorder (2, 3).

The prevalence of ADHD in this study in patients with NF1 is high, at 8.8% (95% CI: 7.2-10.7%), which is significantly (p<0.05) higher than the 5% figure often cited for the prevalence of ADHD, suggesting a significant association with ADHD at the diagnostic level. This study highlights the importance of early diagnosis and treatment of ADHD in patients with NF1 (3).

Our patient was diagnosed with NF1 and ADHD and had a favorable outcome. It is important to bear in mind the combined prevalence of both disorders for early diagnosis and multidisciplinary treatment.

**Disclosure of Interest:**

None Declared

